# Medical Physics Practice Guideline (MPPG) 11.a: Plan and chart review in external beam radiotherapy and brachytherapy

**DOI:** 10.1002/acm2.13366

**Published:** 2021-08-02

**Authors:** Ping Xia, Benjamin J. Sintay, Valdir C. Colussi, Cynthia Chuang, Yeh‐Chi Lo, Deborah Schofield, Michelle Wells, Sumin Zhou

**Affiliations:** ^1^ Department of Radiation Oncology Cleveland Clinic Cleveland Ohio USA; ^2^ Department of Radiation Oncology Cone Health Greensboro North Carolina USA; ^3^ Department of Radiation Oncology University Hospitals Cleveland Medical Center Cleveland Ohio USA; ^4^ Department of Radiation Oncology Stanford University Stanford California USA; ^5^ Department of Radiation Oncology Mount Sinai Hospital‐ New York New York New York USA; ^6^ Department of Radiation Oncology AdventHealth Orlando Orlando Florida USA; ^7^ Department of Radiation Oncology Piedmont Healthcare Atlanta Georgia USA; ^8^ Department of Radiation Oncology University of Nebraska Medical Center Omaha Nebraska USA

**Keywords:** MPPG 11.a, plan and chart review, safety and quality

## Abstract

A therapeutic medical physicist is responsible for reviewing radiation therapy treatment plans and patient charts, including initial treatment plans and new chart review, on treatment chart (weekly) review, and end of treatment chart review for both external beam radiation and brachytherapy. Task group report TG 275 examined this topic using a risk‐based approach to provide a thorough analysis and guidance for best practice. Considering differences in resources and workflows of various clinical practice settings, the Professional Council of the American Association of Physicists in Medicine assembled this task group to develop a practice guideline on the same topic to provide a minimum standard that balances an appropriate level of safety and resource utilization. This medical physics practice guidelines (MPPG) thus provides a concise set of recommendations for medical physicists and other clinical staff regarding the review of treatment plans and patient charts while providing specific recommendations about who to be involved, and when/what to check in the chart review process. The recommendations, particularly those related to the initial plan review process, are critical for preventing errors and ensuring smooth clinical workflow. We believe that an effective review process for high‐risk items should include multiple layers with collective efforts across the department. Therefore, in this report, we make specific recommendations for various roles beyond medical physicists. The recommendations of this MPPG have been reviewed and endorsed by the American Society of Radiologic Technologists and the American Association of Medical Dosimetrists.

**Table:** 

Table of Contents
1. Introduction
Scope and Charges
2. Definitions and Acronyms
3. Expectations, Responsibilities, and Plan/Chart review items
3.1. External Beam Radiation Therapy (EBRT)
3.1.1. Simulation
3.1.2. Prescription and treatment volume delineations
3.1.3. Plan documentation and self‐check items for planners
3.1.4. Initial plan/chart review items for medical physicists
3.1.5. Initial chart review items for radiation therapists
3.1.6. Weekly chart review for EBRT
3.1.7. End of Treatment (EOT) chart review for EBRT
3.2. Brachytherapy ‐ HDR
3.2.1. HDR prescription and treatment volume delineations
3.2.2. HDR documents
3.2.3. Initial plan/chart review items for medical physicists
3.2.4. Initial plan/chart review items for radiation therapists
3.2.5. Weekly chart review for Brachytherapy
3.2.6. End‐of‐Treatment (EOT) chart review for Brachytherapy
4. Computer‐aided Plan/Check and Automation
5: Recommendations and Discussion
5.1. General Recommendations
5.2. Comparison of TG 275 and MPPG 11.a.
6. Summary
References:

## 1 INTRODUCTION

The American Association of Physicists in Medicine (AAPM) Task Group (TG) 40 report,^1^ published in 1994, established the foundation of comprehensive quality assurance (QA) for radiation therapy. Most current medical physics practices in radiation therapy stem from this report. With the advancement of radiation therapy over the past two decades, numerous additional QA reports have been published,^2^, ^3^, ^4^ each addressing one specific aspect of the topics covered in TG 40. Based on a survey of AAPM members and subsequent failure mode and effect analyses (FMEAs), the recently published TG 275^5^ provided recommendations for reviewing radiation therapy treatment plans and patient charts. As defined by AAPM, medical physics practice guidelines (MPPGs) are intended to provide the medical community with a clear description of the minimum level of medical physics support that AAPM would consider to be prudent in all clinical practice settings. Therefore, this MPPG is not designed to emulate TG 275 but rather to provide practice guidelines regarding when, who, what, and how to conduct treatment plan and patient chart review to ensure a minimum standard of physics support is followed.

Radiation therapy involves a coordinated team effort. The process and responsibilities for different team members, for example, radiation oncologists, medical physicists, dosimetrists, radiation therapists, and nursing staff, are clearly defined in “Safety is No Accident,” the white paper from the American Society of Radiation Oncology (ASTRO).^6^ This MPPG focuses on plan and chart QA during treatment preparation and delivery. Depending on the clinical importance, risk, and achievable workflow, some parameters in patient plans and charts may be successively checked by multiple persons from different teams (or multiple times) while others may be checked only once. The purpose of having a patient chart reviewed by different radiation team members at multiple time points throughout the treatment course is to ensure accurate and precise radiation treatment planning and delivery.

A radiation therapy patient chart typically consists of numerous documents and records, including, but not limited to, diagnostic reports, consultation reports, simulation documents, treatment plan reports, daily treatment records, and on‐treatment visit reports. One of the most critical elements of a radiation therapy patient chart is the treatment plan. To distinguish from the clinical plan (defined in Section 2), this report focuses on the technical aspects of a treatment plan which will hereafter be referred to as a “plan” for simplicity. It is critical that the integrity of a plan be reviewed by a qualified medical physicist (QMP) or their designees to ensure safe and high‐quality treatment.

In a recent publication, 33% of errors reported to the Radiation Oncology Incident Learning System (RO‐ILS) were made during the processes of treatment planning and pre‐treatment review/verification.^7^ Mitigating these hazards is best accomplished by catching errors as far upstream as possible and using multiple levels of plan check/review.^8^ The possibility of an error propagating through to a patient can further be minimized by using a standardized treatment plan report, a post‐planning review by the planner, a subsequent review by a QMP or authorized clinical staff under the supervision of the QMP, and a pre‐treatment review by a radiation therapist. This MPPG provides guidance for these reviews. This report has been reviewed and is endorsed by the American Association of Medical Dosimetrists (AAMD) and the American Society of Radiologic Technologists (ASRT).

### 1.1 Scope and charges

The goal of this MPPG is to provide recommendations on plan/chart reviews in the form of example lists of items to check for medical physicists and other clinical staff. These lists are based on a minimum level of physics support deemed necessary for safe patient care. The charges of this MPPG are:

1. To define the roles of dosimetrists, radiation therapists, medical physicists, and qualified medical physicists as they pertain to the treatment plan/chart review process for external beam radiotherapy (EBRT) and brachytherapy.
2. To define a minimum level of practice support for initial, weekly, and end of treatment (EOT) plan/chart reviews organized in the form of lists.
3. To make recommendations on the timing of the initial, weekly, and EOT plan/chart review.

This MPPG provides specific guidance for common forms of radiotherapy including external beam (delivered with C‐arm linear accelerators) using photons and electrons (excluding proton plans) and high dose rate (HDR) brachytherapy for gynecological treatments. It is not feasible for this MPPG to cover every procedure or clinical workflow, and as such, the medical physicist should lead the effort, in collaboration with other department members, to establish appropriate procedures and documentation for those not covered by this MPPG. Radiation oncologists are ultimately responsible for the quality of an accepted treatment plan, such as reviewing all contours, dose conformity, dose tolerance of each specific organ‐at‐risk (OAR), and dose coverage of the targeted tumor volumes. This MPPG does not expound on how to achieve optimal treatment plan quality (eg, what dose goals are optimal), instead, it focuses on plan integrity and chart accuracy (eg, whether the treatment schedule agrees with the prescription). For a more comprehensive plan/chart review process, readers should refer to TG 275,^5^ which considered survey results from the AAPM community and used a FMEA to determine the highest risk aspects of the process.

## 2 DEFINITIONS AND ACRONYMS

**AU:** Authorized users are radiation oncologists who are authorized to utilize an HDR brachytherapy unit and are required by the Nuclear Regulatory Commission (NRC) and agreement states to be present at the treatment console during HDR brachytherapy treatments.

**AMP:** Authorized medical physicists are defined according to the NRC, which can be found on the NRC website. For agreement states with the NRC, AMPs should be listed on the licenses of facilities or be approved by the local Radiation Safety Committee for a specific procedure that uses radioactive materials.

DOB: Date of birth.

**Chart:** All documents that accompany a patient‐specific radiation treatment, including treatment prescription, treatment plan (clinical and technical plans), treatment delivery parameters, recorded treatment deliveries, and image‐guided radiotherapy (IGRT) images. A patient chart typically resides in an Oncology Information System (OIS). A part of an OIS is a radiation specific electronic medical record system (RO‐EMR), which is sometimes called a Record and Verify System (R&V).

**Clinical Plan:** A radiation oncologist should provide the following in a clinical component of a treatment plan: treatment intent, instructions to the planners on how to create the planning target volume (PTV), if desired, total dose, fractionation scheme, and dose limits to OARs, if they are different from the institutional established standard tolerances, as indicated in American College of Radiology (ACR) guidelines.^9^, ^10^

**EOT Chart Review**: An end‐of‐treatment chart check. A review occurring after radiation treatment has ended.

**Initial Plan/Chart Review**: A new plan/chart check, applied to any plan that is either a new or a modified plan with dosimetric changes.

**Dosimetry Plan (“plan”):** A component of a radiation therapy treatment plan, focusing on technical details of the plan. For example, an EBRT plan would consists of dose information, treatment modality, plan parameters, and dose distributions while a brachytherapy plan would contain dose information, utilized isotope, catheter information, and dwell positions.

**Planner:** The staff member, including dosimetrists and medical physicists, creating and optimizing the plan to meet the clinical goals set by radiation oncologists.

**Prescription:** A written directive for radiation therapy. Following the prescription guidelines of ASTRO,^11^ a radiation oncologist should provide the following minimum information in the written prescription for external beam radiation: anatomic site, a delivery technique including photon/electron, energy, total dose, number of fractions, and the percent isodose line that is used for normalization to a specific point or to a particular volume. If IGRT is applicable, the prescription should specify the alignment method (eg, bone or soft tissue) and the frequency of IGRT applications.

**QMP:** As defined by AAPM Professional Policy 1‐J,^12^ a QMP is an individual who has met academic and training requirements, has been granted certification in a specific subfield(s) of medical physics by an appropriate certification body and is competent to independently provide clinical professional services in therapeutic medical physics.

**QMP designee**: A medical physicist or a certified medical dosimetrist who has demonstrated competency in a specific task and can perform the task under general supervision of a QMP.

**RO‐EMR:** Radiation Oncology Electronic Medical Record system.

**Weekly Chart Review:** An on‐treatment chart check. A chart check performed during the course of radiation therapy treatment to ensure patient care is occurring safely and according to the physician's intent. For a more than once‐a‐day treatment schedule, the weekly chart review will be performed more frequently than once a week. Similarly, if the treatment is not scheduled every day, the weekly chart review will be performed once within every consecutive five fractions, not necessarily within a week.

**Written directive**: A term used commonly by regulatory agencies to describe the radiation prescription, frequently associated with procedures utilizing radioactive materials.

## 3 EXPECTATIONS, RESPONSIBILITIES, AND PLAN/CHART REVIEW ITEMS

### 3.1 External beam radiation therapy

A typical workflow for external beam radiation therapy (EBRT) planning starts with the establishment of a prescription by the radiation oncologist. The prescription is then fulfilled by the process of simulation, delineation of tumor volumes and OARs, and creation of a patient‐ specific plan. Errors in a treatment plan may stem from ambiguities in the simulation order and prescription. In this section, we discuss items that should be checked for each step of EBRT planning and provide recommendations of who and when to check these items.

#### 3.1.1 Simulation

Errors in simulation may propagate to other EBRT processes downstream. A written order for simulation from a radiation oncologist is necessary and is an ASTRO recommendation.^6^ The content of this written order may vary from practice to practice. Typically, a simulation order comprises patient positioning (eg, supine vs. prone), method of immobilization if applicable, planning computed tomography (CT) acquisition protocols (eg, 4D CT, breath‐hold), bolus, prior treatment, and special medical conditions (eg, implant device). Table 1 lists recommended simulation order items that should be considered based on an organization's specific practice and workflow. Table 2 lists items that should be checked by simulation therapists to ensure completeness of the simulation order and the presence of critical clinical documents that indicate diagnosis, stage, treatment site, treatment intent, and the use of a specific clinical protocol. Prior to treatment planning and ideally before simulation, radiation oncologists should complete the initial prescription, providing a specific dose and fractionation regimen. After completion of the treatment plan, the initial prescription can be modified and finalized according to the treatment plan as discussed in the next section.

**TABLE 1:** Example simulation orders from radiation oncologists

Sections	Recommended	Optional
Patient information	Patient name, MRN, and DOB	
	Informed consent completed	
	IED (implantable electronic device)/pregnancy/prior RT treatment (Y/N, then choose from a list)	
	Treatment intent (urgent, palliative, definitive, etc.)	
	Patient weight over couch weight limit per local policy (Y/N)	
Treatment site	Anatomic location	
	Bolus needed at simulation	
Patient positioning	Supine, prone, lateral decub, frog leg, etc.	
	Immobilization device (choose from a list)	
	Adaptive planning (eg, resim, use an old isocenter, change immobilization device, etc.)	
	Wire markers at simulation	
	Special shielding required (Y/N)	
	Contrast (oral, IV, urethra, etc.)	
	Organ preparation during simulation (full bladder, empty rectum, etc.)	
Imaging technique	CT scan extension (super‐infer borders)	MRI and other image modality needed
	Breath‐hold, 4DCT	Image fusion needed
Treatment techniques	photon, electron	
	3D (SSD, AP/PA, tangent, etc.), IMRT, SBRT	Treatment delivery system (choose from a list)
IGRT	IGRT method (choose from a list)	
	IGRT alignment method (bone, specific organ, or markers)	

**TABLE 2:** Example checklist items for simulation therapists

Verify Patient name, MRN, and DOB
Verify patient pregnancy status, Implanted electronic device (IED)
Verify Informed consent completed and presence of signatures
Verify treatment site and laterality consistent with informed consent form
Verify presence of diagnosis document that state diagnosis, stage, treatment site, treatment intent, clinical protocol, etc.
Simulation order completed

#### 3.1.2 Prescription and treatment volume delineations

Prescription and treatment volume delineations play vital roles in treatment planning and are the sole responsibility of a radiation oncologist. In keeping with prescription guidelines from ASTRO,^11^ the dose prescription should clearly indicate the treatment anatomical site name and laterality, treatment modalities (eg, photon or electron), total dose, number of fractions, and the method of dose normalization, along with special motion management/image guidance method and any specific clinical considerations (eg, pacemaker).

According to ACR practice guideline,^9^ a radiation oncologist is responsible for the delineation of the tumor volumes, including the gross tumor volume (GTV), clinical target volume (CTV), and internal target volume (ITV). A radiation oncologist may delegate, with clearly written instructions, the responsibility of generating PTVs, and outlining OARs to the planners. However, radiation oncologists are responsible for reviewing and approving all target volumes and OAR contours prior to plan approval.^13^ Any OARs that are not clearly discernible on the planning images should be delineated by the radiation oncologist. The radiation oncologist should also provide detailed written instructions to the planner about the desired dose‐volume coverage to the targeted volumes and dose limits to clinically relevant OARs.^13^ In the case of standard treatments, this can be accomplished via established protocols or scorecards. However, for atypical treatments (eg, re‐treatments), the radiation oncologist should provide patient‐specific dose constraints. Due to limited space or limits in text characters, it may be impractical to include dose constraints or clinical goals in the treatment prescription, but these should be documented elsewhere in RO‐EMR. Tables 3 and 4 list the key items to be included in the prescription for EBRT plans and brachytherapy plans, respectively. If the clinical goals cannot be met during treatment planning, the planner should discuss with the radiation oncologist to determine a reasonable compromise and document the change in the RO‐EMR. If the prescription is modified during treatment, the radiation oncologist should document the change in the RO‐EMR.^14^

**TABLE 3:** Example items included in the prescription for EBRT plans

Recommended	Optional	
Treatment site (anatomic site and laterality)		
Technique (eg, VMAT), modality (eg, photon), energy	
Fractional dose/fraction pattern, total dose		
Normalization points or volume (isodose line (%), absolute dose, treatment depth, specified point, or DVH endpoint)		
CBCT/Imaging technique, frequency, pattern, and alignment structure (daily, weekly, etc.)	Action level after IGRT (if applicable)	
Special motion management (eg, breath‐hold, gating)	
Pre‐RT preparation (eg, full bladder) or other unique situations (eg, pregnant patient, bolus/compensator, prior treatment, concurrent chemo, implant electronic device, in‐vivo measurement, etc.)	
Time interval for non‐standard fraction pattern	
Physician approval (prior to treatment)		

**TABLE 4:** Example items inluced in the written directive for brachytherapy plans

Recommended	Optional	
Treatment site (anatomic site and laterality)		
Applicator name (size, model, and number of catheters if applicable)/isotope	
Fractional dose/fraction pattern, total dose		
Normalization points or volume (isodose line (%),absolute dose, treatment depth/length, specified point, or DVH endpoint)		
Image guidance (if applicable)		
Time interval for non‐standard fraction pattern		
Physician approval (prior to treatment)		

#### 3.1.3 Plan documentation and self‐check items for planners

The planner, often a dosimetrist or a medical physicist, is responsible for creating a treatment plan in accordance with the prescription and other written instructions provided by a radiation oncologist. To avoid potential re‐planning and patient treatment delays, planners should check the consistency and completeness of target volumes and OARs prior to planning. The location and coordinates of the isocenter of the planning CT should be confirmed against the isocenter marked on the patient at simulation (eg, using photos taken at the simulation) and simulation documentation. Additional recommendations for items that planners should check are listed in the first section of Table 5.

**TABLE 5:** Example planner checklist items for EBRT plans

	Recommended	Optional
Planning	Patient identification and correct planning data set Isocenter or reference origin agree with simulation document Treatment site, laterality, and intended dose regimen in Rx agree withsimulation or other documents. CT image adequate (eg, FOV) Couch setting correct (eg, removal/insert table model) Density overrides in contours reasonable Normal/critical structure contours reasonable PTV and ITV are logical (without stray voxels) Dose grid size include all critical contours Bolus documented following local convention (if applicable) Field name/ID correct and following local convention Warning /error messages addressed	Calculation algorithm/resolutions set correctly, particularly for a structure with small volume in SRS Necessary new calculation/reference point added (if applicable) Composite plan if multiple CTs, sequential treatments, or retreatment Use the scheduled treatment machine
Plan document	Planned Rx matched with Rx in RO‐EMR Isodose distributions DVHs Scorecard/DVH metrics meet clinical requirements or clinical protocols (if applicable) Electron/bolus skin renders (if applicable) DRRs with beam shapes are appropriate (3D plan only) Collision check (gantry, couch, and patient body)	Include an Isocenter image slice Display beam configuration in 3D view Follow local documentation standard
RO‐EMR Preparation	Document Isocenter shifts and bolus Additional patient setup instruction following local convention Image guidance/motion management (KVCBCT, MVCBCT, DIBH, etc.) documented Rx approved by physician in RO‐EMR or in Plan Reference CT (isocenter/structures) for CBCT sent (for third party image guidance system) Block/accessory code (eg, electron code) checked Dose tracking parameter set Field parameters set and completed (table vertical, tolerance table, or default table position, SID) Treatment delivery pattern/schedule set correctly DRR associated and set to Tx (for third party RO‐EMR system) SSDs documented	Check number limits of contours/CT slices on IGRT systems Backup timer check

Abbreviations: DIBH, deep inspiration breath hold; DRR, digitally reconstructed radiograph; KVCBCT, Kilo‐voltage cone beam CT; MVCBCT, Mega‐voltage cone beam CT; Rx, prescription; SID, source‐imager distance; SSD, source‐skin distance; Tx, treatment.

Upon completion of the plan, a plan report is typically created, which is ideally stored in a file format, such as PDF, that cannot be easily modified after creation. Each institution should establish a local standardized format for the treatment plan report and adhere to the guidelines from Task Group 262 (publication pending), which is charged with establishing recommendations on the implementation of a RO‐EMR. As stated in TG 262, treatment plan documentation should be easily accessible and serves as an efficient means of communicating with outside institutions upon request. The treatment plan report should be designed to include the necessary information for plan review by physics (weekly checks, end of treatment checks), therapists, and physicians (eg, chart rounds, status check). Depending on the complexity of a plan and details of a plan report, we recommend medical physicists review treatment plans directly within the treatment planning system during initial plan review, particularly if a question about the plan arises.

In addition to being easily accessible, the plan report should provide a durable record of the plan, independent of the planning system, in the event the planning system and/or record and verify data become inaccessible or in parts of the workflow that include groups such as radiation therapists who may be less familiar with all of the features of a TPS. The treatment plan report can also be used as the document of prior treatment(s) in the re‐irradiation setting. In the plan report, the critical plan parameters can be easily archived, documented in secondary or hospital EMR systems, or transferred to other institutions. While DICOM planning data transfer between institutions or planning systems in the re‐irradiation setting is recommended, this may not always be possible due to treatment planning systems becoming obsolete/decommissioned, technology limitations between institutions, or incompatible formats. Additionally, quick access to these data may be vital in the emergent re‐irradiation setting. For these reasons, the members of this MPPG are in a consensus agreement to recommend creating a plan report for each plan to mitigate the hazards of relying solely on proprietary treatment planning data as a durable record of a plan. However, it is recognized that there may be alternative approaches without creating a plan report, specifically, as technology changes or in the circumstances not considered by the MPPG members as a part of this review.

If the RO‐EMR has a separate database from the treatment planning system, the planner is often responsible for transferring the treatment delivery parameters to the RO‐EMR. The information that is transferred may include additional shifts from the initial isocenter (or reference point) set during simulation, modification of patient‐external contours (eg, add a bolus on specific region), all key treatment parameters for each field, and images (DRRs, reference images) associated with the treatment isocenter. This information is essential for radiation therapists to perform accurate patient positioning and treatment delivery.

To facilitate the chart checks (eg, initial and weekly chart checks) conducted by various clinical team members, an example of recommended components in a plan report is listed in Table 6. We recommend that a treatment plan report should include the dose prescription, beam parameters, and dose distributions. Nomenclature, such as beam names, should be standardized and designed to reduce confusion and improve safety. We also recommend organ and tumor volume names follow the standard nomenclature established by TG 263.^15^ If not documented elsewhere, patient setup information should be included in the treatment plan report. When implementing new treatment modalities, the recommended components of the treatment plan report should be revised accordingly by a QMP.

**TABLE 6:** Example EBRT plan report elements

Section	Recommended	Optional
General	Hospital/location Print date or date of service Planning system (version)	Page numbers Plan creation/revision date Planner/staff
Demographics	Patient name/MRN	Date of birth/gender
Prescription/Written directive on plan document	Target Anatomic Site Dose Fractionation Prescription method/plan normalization method	Course/diagnosis identifier Planner/physician approval/date
Plan Summary	Machine identifier Energy, photon/electron Beam names/IDs Gantry angles Collimator angles and sizes RX and normalization MUs per beam Couch angles	
Additional Plan info	Isocenter location Patient or couch shifts Planning CT date/scanner ID Patient orientation (head first/supine) Ref. points/points of interest with location/dose/type	Name of CT density table Import log Plan UID Composite plan information IEC convention
Dose calculation	Method (eg, convolution, AAA, Monte Carlo, etc.) Normalization method Heterogeneity corrections (Y/N) Grid resolution/size Tissue density override Warning messages	
DRRs/Beams eye views (For 3D treatment fields)	Wedge direction in graphical display Patient orientation Beam ID and direction Beam shapes (jaw and MLC) with scale Target contours Critical OAR contour(s) Bolus placement with skin render	
Images with Isodose	Absolute isodose lines with selected target and OARs contours Prescription isodose level(s) Isocenter point or its location Location of Maximum dose or hot spots Patient orientation Slice number	
DVHs (when appropriate)	Structure names Defined dose goal to each structure (Volume, minimum dose, maximum dose, mean dose, etc.) DVHs	

Abbreviations: AAA, Anisotropic analytical algorithm; DVHs, dose‐volume histograms; MRN, Medical record number; OARs, Organs at risk; UID, Unique identification number.

A critical task of a planner is to communicate important details related to the plan setup and execution with radiation therapists. A planner should assemble or update necessary information which may include patient setup parameters, including isocenter shifts, IGRT reference images, bolus placement, and cross‐verification parameters (eg, source to surface distances (SSDs), table positions, etc.). We recommend that the planner conduct a self‐check during planning or after the plan is completed following Table 5. Table 5 also contains items that are important for radiation therapists to check for the safe treatment of patients.

For brachytherapy plans, we do not create a separate table of check items for planners since the HDR plan planning and the treatment console are well integrated and typically the same team member involved in planning is present during treatment. We thus recommend using the same table (Table 7, see below) for planners and for physicists who conduct the secondary check.

**TABLE 7:** Example initial brachytherapy plan check items for medical physicists and their designees

	Recommended	Optional
Planning	Rx matches plan	
	Source activity/air kerma strength (against decay table)	
	Correct planning image (eg, correct date/time)	
	Plan normalization	
	Channel number assignment follow local convention	
	Index length/offset	
	Catheter orientation (tip vs. connector ends)	
	Number of dwell positions and location of the first dwell point	
	Step size	
	Applicator name, size, and model	
	Magnified catheter reconstruction in 3D view	
	Plan optimization appropriate	
	Dose distribution appropriate	
	Calculation algorithm	
	Secondary calculation	
RO‐EMR preparation	Rx and plan approved by physician	
	Plan approved by physicist	

#### 3.1.4 Initial plan/chart review for medical physicists

Medical physicists play an important role in initial plan/chart review, ensuring integrity, accuracy, and clarity. We recommend that a QMP, or a medical physicist under the general supervision of a QMP, complete the initial plan/chart review prior to the first fraction of the plan. During the initial plan/chart review, the medical physicist should ensure that the prescription follows the guidelines (Table 3) and ensure the fractional dose and total dose in the prescription agrees with the treatment plan. Additional recommendations for the medical physicist initial plan/chart review are listed in Table 8. For a solo QMP who also acts as the planner, we recommend that a certified medical dosimetrist conduts the initial plan check by independently reviewing the plan, provided the QMP reviews and approves the final documentation, and performs a secondary MU/dose calculation using a secondary method other than the TPS. Given different practice workflows and available resources, each institution should perform independent assessments of the best methods to catch errors upstream and to avoid treatment delays. Medical physicists should participate in designing an optimal workflow that can catch errors as early as possible in the treatment planning process.

**TABLE 8:** Example initial EBRT treatment plan/chart check items for medical physicists

Sections	Recommended	Optional
Plan integrity check		
	Patient name/MRN/DOB	
	Isocenter/initial reference point matched with simulation doc	Dose calculation algorithm (per institutional policy)
	Isocenter/initial reference point matched with the patient skin marks	
	Isocenter shift documented	
	Rx dose in plan matched with Rx in RO‐EMR	
	Field parameters (MUs, dose, field size, collimator, gantry, and MLC positions) reasonable or following local policy	
	Calculation dose grid included all critical contours	
	Beams associated with appropriate isocenter	
	If multiple isocenters are used, clearly labeling of each isocenter	
	3D field shapes appropriate for physician intent (3D plans only)	
	Plan quality and dose metrics reasonable (if applicable)	
	Beam name following institutional convention	
	Beam modification (bolus) noted and documented	
	Check Beam clearance (potential collision)	
	Correct CT dataset used	
	ROI density override appropriate	
	Deliverability of beams (minimum MU for EDW, and maximum MU allowed for high dose, or high dose box checked, errors and warning messages addressed)	
	Couch included/excluded correctly	
	Implanted electronic device dose documented (if applicable)	
	Prior treatment dose added and plan sum appropriate (if applicable)	
	Report conformal index (SRS/SBRT plan only)	
	Secondary dose calculation difference <5%	
Preparation in RO‐EMR		
	Rx in RO‐EMR in accordance with Table 3	Setup beams associated with the same treatment isocenter
	Rx approved by physician	Image fusion/registration completed and documented
	Plan approved by physician and physicist (or in plan document)	Patient‐specific QA reviewed
	All field parameters input (or associated) correctly and approved (if using third party system)	Special physics consult documented (if applicable)
	Site setup instruction (treatment positions, bolus, motion management, etc) are set correctly	Planned for a scheduled treatment machine
	Reference CT input with correct isocenter and include relevant targets and ROIs	Immobilization appropriate
	DRRs associated and approved (if use a third party system)	Dose tracking point (volume) matched Rx in RO‐EMR
	Tolerance table set correctly	Created QCL/task for therapy check
	CBCT/IGRT alignment instruction presence	
	Treatment schedule (eg, daily, BID) is in agreement with Rx	
	Dose tracking set correctly (if applicable)	
	Special instructions as needed (eg, surface guided imaging parameters, breath‐hold threshold)	

Abbreviations: DIBH, deep inspiration breath hold; DOB, date of birth; DRR, digitally reconstructed radiograph; EDW, enhanced dynamic wedge; IGRT, image‐guided radiotherapy; KVCBCT, Kilo‐voltage cone beam CT; MU, monitor unit; MVCBCT, Mega‐voltage cone beam CT; QCL, quality‐check‐list; RO‐EMR; Radiation specific electron medical record system; ROIs, regions‐of‐interest; Rx, prescription; SID, source‐imager distance; SSD, source‐skin distance; Tx, treatment.

When applicable, the isocenter or the initial reference point marked during CT simulation must agree with the skin marks on the patient since this is the starting point of treatment delivery. A recent publication^14^ from Radiation Oncology Incident Learning System (RO‐ILS) stated that 15% of reported events included a wrong isocenter. We recommend the medical physicist verify that the initial isocenter (or reference point) matches the isocenter recorded in the simulation. The medical physicist should ensure that essential information from the treatment plan report has been correctly transferred into the RO‐EMR and is approved by the assigned team members. Treatments shall not proceed if the approval status is revoked.

For emergency treatments occurring after hours, we recommend that the on‐call medical physicist reviews the treatment plan remotely or in‐person. For institutions that do not have an on‐call physicist, a radiation oncologist may conduct the initial plan/chart review and the QMP or QMP‐designated medical physicist should check the plan on the next business day, or prior to the treatment on the next business day if additional fractions are prescribed.

#### 3.1.5 Initial chart review items for radiation therapists

Radiation therapists are responsible for positioning the patient for treatment according to the setup instructions and the treatment plan. As an additional layer of safety, it is important for radiation therapists to conduct an initial chart review to confirm that critical treatment parameters and information are available, approved, and consistent prior to any new treatment. The importance of radiation therapists conducting an initial check is well demonstrated.^16^ Recommendations for radiation therapist initial plan/chart reviews are listed in Table 9. For critical items, such as the prescription and location of the isocenter, the initial plan/chart check review items for the radiation therapist intentionally overlap with those items listed on the initial plan/chart checked by the medical physicist. Furthermore, radiation therapists verify the presence and completion of important clinical documents (eg, informed consent form) according to the guidelines published by accrediting bodies such as NRC and other regulatory agencies.

**TABLE 9:** Example initial treatment plan (EBRT) check items for radiation therapists

Recommended	Optional
Patient name, MRN, DOB	Enter “custom” for Bolus fields
Patient photos and setup photos	DRR approved and associated
Consent signed by physician	MUs/daily dose reasonable
Rx signed by physician	Peer review signed by physician/physicist
Plan laterality matched with Rx in RO‐EMR	Treatment couch inserted in plan
Plan used the scheduled treatment unit	Intra‐treatment image method (eg, triggered imaging) in RX
Isocenter shifts instruction clear and documented in setup note	Fiducial contoured
Rx dose/fractions in RO‐EMR agree with plan	Check corrected images input to the third party image systems
Rx Tx technique in RO‐EMR agrees with plan	
Rx energy in RO‐EMR agrees with plan	Reference CT/scan sent to third party system
Field parameters consistency between plan and RO‐EMR (for third party RO‐EMR)	ROI box drawn appropriately
SSD parameters	Bolus field entered
Treatment fields approved by physicist	
Treatment plan deliverability check (loading reference CT and collision check for non‐coplanar beams)	Physics consult presence
Patient setup (immobilization device setup, skin markers) info verified	Dose rate
Special alerts/dose actions (position, bolus, in‐vivo measurement, etc.)	Blocks/compensator in Rx and setup note
Bolus documented	
In‐vivo measurement indicated	
Respiratory/IGRT instructions	
Third party IGRT data ready	
Motion management parameters	
Treatment schedule	
Boost fields are scheduled/follow‐up	
Tx appointment conflicts, concurrent chemo documented	
Implanted device presence/document	
Second MU check documented	
Physics check completed/approved	
IMRT/VMAT QA completed/approved	
Pre‐RT preparations	
*For electron fields:*	
e‐applicator field parameters (energy cone, field size, gantry)	
e‐block checked to the template	
e‐block code verify (accessory code)	

Abbreviations: DIBH, deep inspiration breath hold; DRR, digitally reconstructed radiograph; EDW, enhanced dynamic wedge; IGRT, image‐guided radiotherapy; KVCBCT, Kilo‐voltage cone beam CT; MU, monitor unit; MVCBCT, Mega‐voltage cone beam CT; QCL, quality‐check‐list; RO‐EMR, Radiation oncology specific electron medical record system; ROIs, regions‐of‐interest; Rx, prescription; SID, source‐imager distance; SSD, source‐skin distance; Tx, treatment.

#### 3.1.6 Weekly chart review for EBRT

The weekly chart review typically examines treatment delivery data, including overrides, incomplete treatments, isocenter, or treatment couch shifts after the initial or daily image verification, status of verification image approval (whether or not the verification images were approved by a radiation oncologist), and documentation of patient positioning such as SSDs. While the review and approval of on treatment imaging is solely the responsibility of the radiation oncologist, the medical physicist should review and ensure that frequency, modality (eg, kV vs. MV ports), and magnitude of isocenter shifts under image guidance are consistent with the written directive and in accordance with institutional procedures. Table 10 lists recommended chart check items during weekly chart review. After completion of the weekly chart review, ideally, a report based on the check items in Table 10 should be generated. On the first weekly chart check, we recommend including a second review of the treatment plan by opening the plan report or opening the plan in the treatment planning system to ensure no gross errors omitted by the initial plan review. On the first weekly chart check for IMRT plans, we also recommend checking whether IMRT QA and documentation are completed prior to the first fraction of the treatment. Any change that affects the dosimetry of a treatment plan should be handled as a new treatment plan, and a new report of the modified plan should be created. For example, a change in daily fraction dose requires a new report to document the change to monitor units (MUs). The modified plan should undergo an initial plan/chart review. If the number of fractions of a plan is changed by the radiation oncologist without changing the daily fraction dose, this plan does not need to go through a new plan check process, but appropriate documentation is required.

**TABLE 10:** Example weekly chart review items for EBRT plans

Recommended	Optional
Rx site	Daily prior treatment timeout documented
Rx changed or field modified since last check (updated document or added comment)	CBCT and portal images approved
Dose delivered to date	
Number of fractions delivered	Correct tolerance table applied
Plan quality reasonable (applied to the first weekly check for each plan)	Bolus fields are indicated in setup note
IMRT QA done and approved (applied to the first weekly check)	
Image frequency and modality agree with Rx	Treatment calendar is correct
Dose tracking correct	Review rejected IGRT images
Overrides with proper comments	
In‐vivo measured required and results documented	
Review journal entries/patient notes	
Treatment breaks documented	
Special device or medical condition (pacemakers, etc.)	
Secondary setup verification documented and within limits where applicable (eg, SSDs, SGRT, separation)	
Couch parameters and IGRT shifts within limits or have a note	

Weekly chart reviews should be completed within every five treatment fractions or before the next block of five treatments begins. For a more than once‐a‐day treatment schedule, the weekly chart review will be performed more frequently than once a week. Similarly, if the treatment is not scheduled every day, the weekly chart review will be performed once within every consecutive five fractions, not necessarily within a week. For a non‐conventional treatment schedule with less than five fractions, the institution should consider developing a process to ensure at least one weekly chart review is conducted during the course of treatment, ideally near the beginning of the course. For single fraction treatments, a weekly chart check may be omitted, but the EOT chart check should be reviewed according to section 3.1.7. Weekly chart reviews should be completed by QMPs or QMP‐designated medical physicists, who are under the general supervision of a QMP. The QMP may designate a dosimetrist to assist with the weekly chart check on a rotating basis. If a dosimetrist assists in the weekly chart check, we recommended a medical physicist review and approve the weekly chart check documentation. It is recommended, when possible, that QMPs and/or their designees conduct the weekly chart reviews on an alternating basis, so that the same person does not conduct the weekly chart check for the entire treatment course.

#### 3.1.7 End of Treatment (EOT) chart review for EBRT

The purpose of an EOT chart review is to ensure that the treatment prescription has been fulfilled accurately and all pertinent documents are presented and approved. The EOT chart review should be completed by the QMP and/or their designed medical physicists within five business days of the patient’s last delivered fraction.^17^ For a single fraction treatment course, ideally, the EOT chart review should be conducted on the same day of the treatment or on the next business day, no later than five business days. The recommended items to be checked during the EOT are listed in Table 11. If the prescribed treatment course is not completed, a comment that clearly documents the aborted treatment should be added to a highly visible location in the chart such as the existing prescription, on a separate note, or in the EOT chart review document.

**TABLE 11:** Example end of treatment chart review items

Recommended	Optional
Treatment Site	
Total dose delivered	Are all weekly checks done and appropriate
Number of fractions delivered	All verification images reviewed
Total dose delivered agrees with Rx (if not, proper documentation in the medical record)	
All documents signed (except for completion note)	

#### 3.1.8 Brachytherapy ‐ HDR

Brachytherapy encompasses a broad scope of procedures making it infeasible to cover each situation with this report. This MPPG examines a common high dose rate (HDR) brachytherapy for gynecological cancer. The plan/chart check recommendations for this procedure may be extended to other more complicated brachytherapy treatments or procedures under the guidance of an authorized medical physicist (AMP).

#### 3.1.9 HDR prescription and treatment volume delineations

Similar to EBRT, the HDR prescription and treatment volume delineations are solely the responsibilities of radiation oncologists. According to the prescription guidelines from ASTRO,^11^ the dose prescription should clearly indicate the treatment anatomical site name and laterality, isotope used for treatment, total dose, number of fractions, and the method of dose normalization (eg, DVH volume‐dose normalization) or the treatment depth. Specific to brachytherapy, we recommend including items listed in Table 4 such as applicator size and name (or model). With clearly written instructions, the radiation oncologist may delegate the responsibility of PTV generation to a planner. However, the radiation oncologist is responsible for reviewing and approving all target volume and OAR contours. The radiation oncologist should also establish planning guidelines for the planner by specifying the desired dose‐volume coverage to the targeted volumes and dose limits to clinically relevant OARs (sometimes referred to as dose constraints or clinical goals).^13^

#### 3.1.10 HDR documents

The brachytherapy treatment plan report should be similar to EBRT. Table 12 shows the various components of a brachytherapy plan report. It is recommended to include the reconstruction of each catheter and/or source position(s), including orientation (eg, the tip vs. connector end of the catheter). It should be noted that the verification of the reconstruction may not be entirely possible using a report alone and likely requires a review within the treatment planning system (TPS).

**TABLE 12:** Example brachytherapy treatment plan report elements

Section	Recommended	Optional
General	Hospital/location Print date or date of service Planning system (version)	Page numbers Plan creation/revision date Planner/staff
Demographics	Patient name/MRN	Date of birth/gender
Prescription/Written directive on plan document	Target Anatomic Site Dose Fractionation Prescription method/Plan normalization method	Course/diagnosis identifier Planner/physician approval/date
Plan Summary	Isotope Initial calibration (date, time and source strength) current activity and decay factor Catheter connection channel identifiers First dwell position and offset Catheter (length/index) Source positions (step/space) and dwell time Total delivery time	
Additional Plan info	Treatment date and time Ref points/points of interests Treatment unit name Applicator name, size	
Dose calculation	Calculation method (eg, TG−43, TG−186, etc.)	
Catheter 3D View	Catheter reconstruction view Applicator model name used for planning	
Images with Isodose	Absolute isodose lines with selected target and OARs contours Prescription isodose level(s) Patient orientation	
DVHs	Structure names Defined dose goals to each treatment volume and OARs DVHs	

#### 3.1.11 Initial plan/chart review items for medical physicists

The initial plan/chart review for HDR should be performed by an AMP. In situations where an AMP is in solo practice, an AMP‐designated team member (such as a radiation therapist, dosimetrist, or AU, who is specially trained in the HDR procedure) may complete an independent initial plan/chart review if the HDR plan was created by the solo AMP.

During HDR planning the accuracy of image co‐registration, applicator specification and placement, catheter reconstruction, and catheter channel parameters (such as index lengths and the appropriate identification of the first dwell position) is important parameters to verify before transferring the treatment data to the treatment console. Table 7 lists the recommended items to be checked.

#### 3.1.12 Initial plan/chart review items for radiation therapists

Prior to each treatment, we recommend the radiation therapist, or the AMP if a radiation therapist is not involved in the procedure, to conduct a pre‐treatment check following Table 13. In the latter scenario, AU should deliver the HDR treatment. The check items listed in Table 13 can be served as a part of the treatment procedure document. Both the AMP and AU who are present during the treatment should approve the procedure document.

**TABLE 13:** Example brachytherapy pre/post‐treatment check items

	Recommended	Optional
Pre‐treatment	Patient identification with two methods	Patient consent form signed
	Rx matched with plan and approved by an physician	Review setup photos
	Plan approved by physicist	Documentation of survey meter
	Correct applicator inserted (size, model)	
	Current source activity (against decay table)	
	Correct plan loaded	
	Total treatment time correct	
	Catheter channel number correct and follow local convention	
	Catheter length/step size correct	
	Patient pre‐treatment survey done	
	Secondary dose check done	
	Daily QA	
	Radiation emergency tools present	
Post‐treatment	Post‐treatment survey	
	Treatment procedure documentation	

#### 3.1.13 Weekly chart review for brachytherapy

Some institutions may generate a new plan for every fraction including an additional initial plan/chart review. In this scenario, the weekly plan review can be omitted. In institutions where the same brachytherapy plan is delivered over multiple fractions, the AMP should complete a weekly chart review within a window of every 5 fractions, similar to those conducted for EBRT. In addition to the weekly plan review, the AMP should review treatment procedure documentation after each treatment. Specific weekly chart check items for brachytherapy are also listed in Table 14.

**TABLE 14:** Example weekly chart review items for brachytherapy plans

Recommended
Rx site
Rx changed or field modified since last check
Dose delivered to date
Number of fractions delivered
Plan quality reasonable
Dose tracking correct
Pre/post‐treatment survey documented

#### 3.1.14 End‐of‐Treatment (EOT) chart review for brachytherapy

Similar to EBRT, the EOT chart review should be performed at the end of the treatment course. The AMP should check the accuracy of all treatment delivery parameters and documents to ensure the AUs’ directives have been fulfilled and that all pertinent technical documents are approved. It is recommended that an AMP completes the EOT chart review on the last day of treatment, or the next business day, but after no later than five business days. The recommended review items for brachytherapy are the same as those for EBRT listed in Table 11.

## 4 COMPUTER‐AIDED PLAN/CHECK AND AUTOMATION

Some vendors and individual institutions have developed computer programs to automatically check various parts of a patient plan or chart.^18^, ^19^, ^20^, ^21^, ^22^, ^23^ These programs are effective in checking logistic requirements and numerical consistency. For example, a computer program can check whether a prescription or portal image is approved by the radiation oncologist^24^ or whether radiation treatment parameters agree with the planned parameters.^23^ A comprehensive literature review of computer‐aided plan/chart check can be found in TG 275.^5^ Due to significant variations in workflow among different practices, these programs cannot completely replace the function of a medical physicist in the process of the plan and chart review. Computer programs, however, can assist in and improve certain areas of plan/chart review. For example, vendor‐provided programs^25^, ^26^ can assist with checking the metrics of plan DVHs against clinical requirements, but currently these programs cannot replace all aspects of a robust plan check such as the careful examination of three‐dimensional dose distributions.

With automation, plan report components recommended in Table 6 can be easily standardized and implemented. Some items listed in Tables 5, 8, and 9 can be checked automatically to avoid upstream errors during treatment planning, but other items such as whether the isocenter marked on the patient skin or a patient mask agrees with the isocenter in a plan may rely on manual examination. Computer‐aided programs can provide alerts of plan/chart review, calling special attention to missed or mismatched items, thereby streamlining the review process and allowing the QMP to focus on manually checked items.

Before implementing computer‐aided chart check programs, it is the responsibility of the QMP to rigorously validate them to avoid potential systematic errors. With the increasing use of artificial intelligence and sophisticated machine learning tools, more solutions are expected to be available clinically in the near future. The combination of computer‐aided and human plan/chart review can significantly improve the effectiveness and efficiency of the plan/chart review process while improving the safety and quality of patient care.

## 5 RECOMMENDATIONS AND DISCUSSION

### 5.1 General recommendations

The primary goal of this MPPG is to provide recommended minimum practice standards for medical physicists and other clinical staff when conducting plan/chart reviews. To maintain diversity and represent the widest range of practices, the MPPG task group included members from academic and community practices using different RO‐EMR (record and verify) systems and treatment planning systems. Given the variations in practice, medical physicists should participate in designing the general workflow to confirm multiple checks are in place for critical documents such as informed consent form, diagnosis, stage, etc. The plan/chart review process, particularly the initial plan review process, is critical to prevent errors and to ensure smooth patient care. The process should have multiple layers of check, including collective efforts across the department to provide adequate and safe chart check/review.

Radiation oncologists are important clinical leaders of the radiotherapy team. It is vital that all orders, instructions, and prescribed parameters are complete and easily understood by all team members throughout the entire process of radiotherapy. We recommend checking the completeness of these orders by providing examples in Tables 1, 2, 3, 4, 5. To catch errors upstream, this MPPG recommends planners conduct a consistency check after completion of a plan.

In accordance with the ASTRO guidelines,^11^ this MPPG recommends that each local institution establish its own standard format of treatment prescription for both EBRT and brachytherapy. The radiation oncologist is ultimately responsible for the prescribed course of therapy. However, medical physicists should be familiar enough with common approaches for major disease sites to determine if a prescription is reasonable or if an obvious error in the prescription has potentially been made. The accuracy and completeness of a prescription are beyond the clinical training and responsibility of a medical physicist.

Depending on the workflow, we recommend each local institution reviews and develops their own standard plan documentation (i.e., plan reports). The standard plan reports can assist non‐physics staff members who may not be familiar with the treatment planning system to grasp the overall plan content. We strongly recommend medical physicists directly review treatment plans within the treatment planning system and not rely solely on the plan document. We also recommend radiation therapists conduct an initial plan/chart review prior to any new treatment.

The scope of this MPPG is limited to common external beam radiotherapy (delivered with C‐arm linear accelerators) and brachytherapy (for gynecological treatments) techniques. The recommendations may be incorporated into other external beam modalities such as proton beam radiotherapy, Cyberknife, Tomotherapy, etc., while considering the unique aspects of these modalities. Institutions that desire to conduct a more comprehensive plan/chart review and have the required resources are encouraged to develop their own procedures combining recommendations from TG 275 and this MPPG while using other sources such as TG 100.^27^

### 5.2 Comparison of TG 275 and MPPG 11.a

TG 275 was referenced by the members of this MPPG when developing recommendations for checking plan/chart integrity.^5^ TG 315, the task group for this MPPG, included two members from TG 275. These two members provided a valuable link for consistency and data sharing between TG 275 and TG 315. The recommendations of this MPPG do not include all of the items from TG 275 as a part of the physics initial plan/chart check—rather we defer some plan/chart check items to dosimetrists (or planners), simulation therapists, and treatment therapists. Further, the recommendations of this report are intended to define a minimum level of support for safe and effective care. Compared to TG 275, this MPPG provides additional descriptions and considers practices with limited resources.

## 6 SUMMARY

This MPPG provides recommendations for medical physicists and other clinical staff to follow in plan and chart review that meet a minimum standard for quality of care. The report also provides key elements that should be considered in plan/chart documentation, minimum professional qualifications for those conducting plan/chart review, and appropriate timelines for completing plan/chart reviews.
